# Development of a Rapid, Accurate, and On-Site Detection Protocol for Red Imported Fire Ants, *Solenopsis invicta* (Hymenoptera: Formicidae)

**DOI:** 10.3390/bioengineering9090434

**Published:** 2022-09-02

**Authors:** A-Young Kim, Young Ho Koh

**Affiliations:** 1 Ilsong Institute of Life Science, Hallym University, Seoul 07247, Korea; 2 Department of Bio-Medical Gerontology, Hallym University, Chooncheon 24252, Korea

**Keywords:** molecular diagnostics, loop-mediated isothermal amplification, species identification, on-site

## Abstract

A rapid, accurate, and on-site molecular diagnostic protocol for red imported fire ants *(Solenopsis invicta*, *Si*) was developed using loop-mediated isothermal amplification (LAMP) assays. *Si*11977 (GenBank accession no. MK986826) was confirmed to be a *Si*-specific gene. Four-primer *Si*11977-LAMP (4p*Si*-LAMP) and six-primer *Si*11977-LAMP (6p*Si*-LAMP) assays specifically detected *Si*. The reaction time of 6p*Si*-LAMP assays was reduced by 5 min compared with 4p*Si*-LAMP assays. The optimal amount of polymerase and the detection limit for the 6p*Si*-LAMP assays were 0.1 unit/μL and 5 fg/μL, respectively. In addition, a method for extracting genomic DNA from ant tissues within 2 to 3 min and a protocol for performing on-site LAMP assays using a car heating mug and a LAMP observation box were described. The on-site *Si* detection protocol used in this study was possible within 30 min from DNA extraction to species identification.

## 1. Introduction

The innovation of transportation has dramatically increased the exchange of people and goods between countries or continents, but it also increases the possibility of the spread of new pests to geographically distant regions [1,2]. The most significant example may be the red imported fire ant [*Solenopsis invicta* Baren (*Si*)], which is one of the world’s 100 worst invasive alien species because of its aggressive behavior, adaptability, reproductive capacity, and survivability [2]. Originating in the Pantanal of South America, *Si* has infested the United States and the Caribbean islands and has now spread to Australia, China, Indonesia, the Philippines, and Taiwan [3]. Recently, *Si* has been frequently found in the international ports of the Republic of Korea and Japan [4].

To prevent invading foreign pests from becoming permanently settled, the removal of alien pests mixed with or hidden in imported goods is essential during quarantine processes at harbors or airports [5,6]. Since the container terminals of the international ports are dangerous areas where many cranes and vehicles are constantly moving to transport numerous imported containers, it is necessary to quickly determine whether or not captured ants from international ports are *Si* during the quarantine process of imported goods. The polymorphism of *Si* in a colony makes it difficult to distinguish *Si* from other native ants using morphological characteristics unless captured ants are examined by ant taxonomists [7,8]. To circumvent these difficulties, various *Si* diagnostics have been developed. Currently available *Si* diagnostic methods include rapid lateral diffusion assays (RLDAs) using species-specific monoclonal antibodies [9,10] and molecular diagnostics using nucleic acid sequence-specific amplification [4,8,11,12]. Although RLDAs allow on-site diagnosis within 30 min, three to five fresh ant samples are needed for reliable diagnosis [9,10]. Because most molecular diagnostics use sequence differences of mitochondrial DNAs, the extraction process of genetic materials from samples and the use of various PCR thermal cyclers are necessary [4,8,11]. Nevertheless, PCR-based molecular diagnostics are highly sensitive and accurate and only require small amounts of samples [4,13]. However, PCR-based molecular diagnostics are more time-consuming compared with RLDAs and are not suitable for on-site diagnosis [4,10,14]. Various nucleic acid amplification methods have been developed to overcome the disadvantages of PCR-based molecular diagnostics. Loop-mediated isothermal amplification (LAMP) is the most widely used method for detecting viruses, microorganisms, nematodes, and insects because it shows outstanding sequence specificity and sensitivity [13,15,16,17,18,19,20,21,22].

LAMP reactions can proceed when a constant temperature between 55 °C and 70 °C is maintained, and assay results can be easily confirmed by adding a double-stranded (ds)-DNA-intercalating fluorophore [16]. Recently, a LAMP assay using the cytochrome c oxidase I (COX1) sequences of the mitochondrial genome of *Si* was reported [12]. Excluding the time to extract *Si* genomic DNA, the *Si*-COX1 LAMP assay required a 90-min incubation time, which is at least three times longer than the time required for commercially available *Si*-RLDA.

In this study, we developed a new *Si* on-site diagnosis protocol for detecting *Si* within 30 min using a body part of *Si*.

## 2. Materials and Methods

### 2.1. Collection of Si and Seven Other Ant Species

*Si* collected from Pusan and Incheon Port in Korea in 2018 was transported to the laboratory and immediately frozen at −50 °C [4]. *Solenopsis geminata* (*Sg*) collected from Vietnam and Laos in 2019 was frozen, imported, and stored at −50 °C. *S. japonica* (*Sj*) and *Pheidole fevida* (*Pf*) collected from Chiak Mountain, Wonju, Gangwon-do were immediately frozen. *Tetramorium tsushimae* (*Tt*), *Lasius niger* (*Ln*), *Formica japonica* (*Fj*), and *Crematogaster matsumurai* (*Cm*) were collected from the central park, Dongan-gu, Anyang, Gyeonggi-do, Korea. All ants used in this study were stored at −50 °C before processing.

### 2.2. Extraction of Genomic DNA from Eight Ant Species

To extract genomic DNA from ant specimens, a DNeasy Blood & Tissue Genomic DNA kit (Qiagen, Valencia, CA, USA) was used per the manufacturer’s protocol. The concentrations of genomic DNA were determined by an epoxy microplate reader (BioTek, Winooski, VT, USA) and then adjusted to 10 ng/μL. Three biological replicates were conducted for each ant species.

### 2.3. Verification of a Si-specific Gene by Conventional PCR

In the previous study [4], we reported two *Si*-specific genes: *Si*11108 and 11977. In this study, we used *Si*11977 to develop a *Si*-specific LAMP assay. First, the specificity of *Si*11977 was confirmed by performing conventional PCR with *Si*11977-forward primer 3 (-F3) and *Si*11977-backward primer 3 (B3) (Table 1).

The 20-μL PCR mixture contained 0.2 μM *Si*11977-F3 and *Si*11977-B3 primers, 1× r-Taq Master Mix (iNtRON Biotechnology, Seong-Nam, Korea), and 0.5 ng/μL ant genomic DNA. The PCR conditions were 95 °C for 3 min, followed by 35 cycles of 95 °C for 30 s, 62 °C for 10 s, and 72 °C for 10 s and one cycle of 72°C for 10 min. PCRs were separated by 2.0% agarose gel electrophoresis (1× TBE buffer).

### 2.4. The 4pSi-LAMP Assay Protocol

To develop a *Si*-specific LAMP assay, *Si*11977-forward internal primer (FIP) and *Si*11977-backward internal primer (BIP) were designed as previously described (Table 1) [4,16]. The 4p*Si*-LAMP reaction mixture contained 1× I buffer 5 (Optigene, Horsham, England), 0.2 unit/μL GspSSD2.0 DNA polymerase (Optigene), 1.6 M betaine (Merck, Kenilworth, NJ, USA), 0.4 mM dNTPs each (Enzynomics, DaeJeon, Korea), 10 mM MgSO_4_ (Optigene), 0.2 M trehalose (DaeJung Chemicals & Metals Co., Ltd., Siheung, Korea), 0.2 μM *Si*11977-F3 and *Si*11977-B3 oligomers, 0.8 μM *Si*11977-FIP and *Si*11977-BIP oligomers, and 0.5 ng/μL ant genomic DNA. After performing 4p*Si*-LAMP assays at 60 °C, 65 °C, and 70 °C, we found that 65 °C was the optimal temperature (data not shown). Because successful LAMP reactions generated large amounts of DNA fragments, these amplified DNA fragments could spread as aerosols and contaminate a laboratory where reaction tubes were open. These contaminants might cause false-positive reactions [14]. Mixing ds-DNA-intercalating fluorophores [e.g., SYBR green I (Thermo Fisher Scientific, Waltham, MA, USA)] with LAMP mixtures extended the incubation times to complete the reactions (data not shown). To avoid these problems, a fluorophore dye-delivering device (Prime4Dia, Anyang, Korea) with 0.45 μL of SYBR green I was placed inside a LAMP tube. After incubation was complete, SYBR green I was mixed with LAMP mixtures in PCR tubes by quick spinning for 2 s at 1000 rpm with a PCR tube microcentrifuge (CF-5, DaeHan Sci., Seoul, Korea). Positive signals were detected by the naked eye under daylight and photographed with a camera in a cell phone (LM-X420N, LG, Seoul, Korea; iPhone 8, Apple, Cupertino, CA, USA). Stronger positive fluorescence signals were detected by a LAMP fluorescence observation box (Prime4Dia) and photographed with a camera in a cellular phone (LM-X420N, LG; iPhone8, Apple).

To determine optimal incubation periods for the 4p*Si*-LAMP assay, we conducted an experiment with a Con and eight 4p*Si*-LAMP tubes with *Si* genomic DNA. The Con tube was incubated for 30 min at 65 °C, and eight tubes were incubated from 12.5 to 30 min at 2.5-min intervals at 65 °C. The fluorescence signals and DNA amplifications were examined as described above.

### 2.5. The 6pSi-LAMP Assay Protocol

Next, 0.4 μM loop forward primer (LF) and loop backward primer (LB) were added to the 4p*Si*-LAMP mixture and incubated at 65 °C. To examine the specificity of a 6p*Si*-LAMP assay, a Con and eight 6p*Si*-LAMP reactions with 0.5 ng/μL genomic DNA from eight ants were mixed and then incubated for 30 min at 65 °C.

To identify the optimal incubation period for the 6p*Si*-LAMP assay, eight tubes with 0.5 ng/μL *Si* genomic DNA were prepared, and reactions were performed at intervals of 2.5-min from 12.5 to 30 min at 65 °C. A Con LAMP tube was incubated for 30 min at 65 °C. The fluorescence signals and DNA amplifications were confirmed as mentioned above.

### 2.6. Determination of Optimal Amounts of LAMP Polymerase

To determine optimal amounts of LAMP polymerase for 6p*Si*-LAMP assays, 6p*Si*-LAMP mixtures containing different amounts of GspSDD 2.0 DNA polymerase were made. Eight tubes containing 0.5 ng/μL *Si* genomic DNA increased the amount of GspSDD 2.0 DNA polymerase from 0.025 unit/μL to 0.2 unit/μL in increments of 0.025 unit/μL. A Con tube contained 0.2 unit/μL of GspSDD 2.0 DNA polymerase.

### 2.7. Detection Limit of 6pSi-LAMP Assay

To determine the detection limit of the 6p*Si*-LAMP assay, 0.5 fg, 5 fg, 50 fg, 0.5 pg, 5 pg, 50 pg, 0.5 ng, or 5 ng of *Si* genomic DNA was added to 10 μL of a 6p*Si*-LAMP mixture. One μL of dH_2_O was added to a Con tube.

### 2.8. The 6pSi-LAMP Assay with a Rapid Genomic Extraction Protocol

The major shortcoming with using LAMP assays in the field is the time-consuming nature of the extraction of genetic material. Fifty μL or 300 μL of Quick genomic DNA extraction solution (QGDES, Prime4Dia) was used to extract genomic DNA from a leg or a body segment from *Si* or *Sg*, respectively. After grinding for 2 to 3 min with QGDES (Prime4Dia), extracted genomic DNA was added to 6p*Si*-LAMP mixtures at a 1:20 ratio without further processing.

### 2.9. On-Site Si Diagnostic Protocol

A protocol using QGDES (Prime4Dia) and 6p*Si*-LAMP assays was established to develop rapid on-site diagnosis technology. For on-site verification, genomic DNA was extracted from an abdomen of *Si* and *Sg* brought from the laboratory, and *Cm* was collected from a rest area on the hillside of Mount Suri, located 150 m above sea level, Anyang, Gyeonggi-do, Republic of Korea by grinding with QGDES for 3 min, followed by mixing with a 6p*Si*-LAMP mixture at a ratio of 1:20. LAMP assay tubes were incubated for 20 min in a portable car heating mug (Red Salmon, Guangdong, China) and then mixed with SYBR green. Species identification was performed based on fluorescence signals using a LAMP-observation box (Prime4Dia) operated with a cell phone portable battery (20,000 mAh, Xiaomi, Beijing, China). 

## 3. Results

### 3.1. Si11977 Was a Si-Specific Biomarker

**Figure 1:**
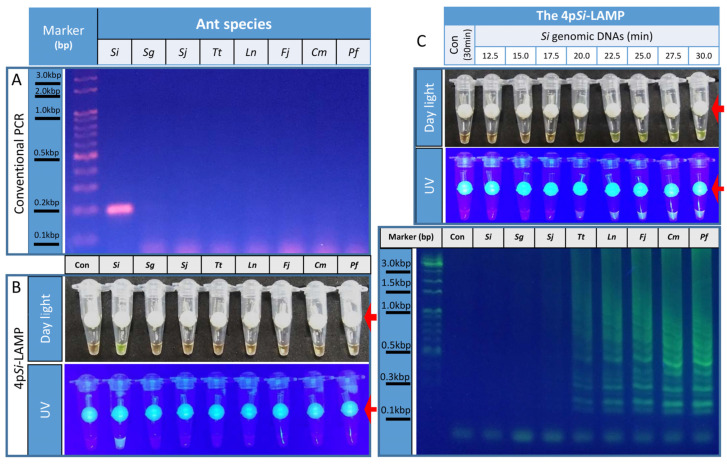
*Si*11977 was a *Si*-specific biomarker. (**A**) The *Si*11977-F3 and *Si*11977-B3 primer pair specifically amplified a DNA fragment from *Si* genomic DNA. A strong 196-bp band was detected from *Si* but not from the other seven ants. (**B**) When the 4p*Si*-LAMP assays were performed, strong fluorescence signals under daylight or UV light were only detected from the tube containing *Si* genomic DNA. (**C**) To determine the optimum incubation period for the 4p*Si*-LAMP assay, LAMP mixtures containing 5 ng of *Si* genomic DNA were incubated at 65 °C for 12.5 to 30 min at 2.5-min intervals. Fluorescence signals under daylight or UV illumination were detected after 20 min. When those reactions were separated by a 1.5% agarose gel, ladder-shaped DNA bands were detected from tubes incubated for longer than 20 min. Red arrows indicate the fluorescence dye-delivering devices, which are white round beads with a short and thin tube in the center.

To verify that *Si*11977 is a *Si*-specific biomarker, conventional PCR was performed with the *Si*11977-F3 and *Si*11977-B3 pairs. When 0.5 ng/μL genomic DNA extracted from *Si, Sg*, or six Korea native ants was used as a template, a 196-bp band was only amplified from *Si* genomic DNA, not from the genomic DNA of other ants (Figure 1A). This suggests that *Si*11977 could be a *Si*-specific biomarker.

### 3.2. Development of a Four-Primer Si-LAMP Assay

Since LAMP assays require at least four primers, *Si*11977-forward internal primer (FIP) and *Si*11977-backward internal primer (BIP) were generated, and a four-primer *Si*-specific LAMP (4p*Si*-LAMP) assay was performed (Figure 1B). To show that there were no false-positive reactions caused by primer dimerization, interconnections among primers, or contamination, a negative control (Con) LAMP without genomic DNA was made. When genomic DNA from *Si*, *Sg*, or six other native ants in Korea was used for 4p*Si*-LAMP assays, strong fluorescence signals were detected only from *Si* genomic DNA-containing tubes (Figure 1B). When tubes were examined under daylight, fluorescence signals from SYBR green I bound to ds-DNAs were observed. Clear fluorescence signal differences among tubes were observed when tubes were placed and observed in a LAMP observation box (Figure 1B). These results suggested that the 4p*Si*-LAMP assay could be used to confirm the identification of *Si*.

### 3.3. Determination of the Optimal Incubation Periods for the 4pSi-LAMP

The optimum incubation period of the 4p*Si*-LAMP assay was determined by incubating LAMP mixtures for different lengths of time. The Con incubated for 30 min did not show any signal (Figure 1C). When LAMP reactions containing *Si* genomic DNA were incubated at 65 °C, weak or strong fluorescence signals from tubes were detected after 20 or 25 min of incubation, respectively, under daylight or UV light by a LAMP observation box. When LAMP reactions were separated by a 1.5% agarose gel, typical ladder-shaped DNA bands were detected after 20 min of incubation, and the patterns and intensities of DNA fragments were similar after 27.5 min of incubation (Figure 1C).

### 3.4. Development of the Six-Primer Si-LAMP

**Figure 2:**
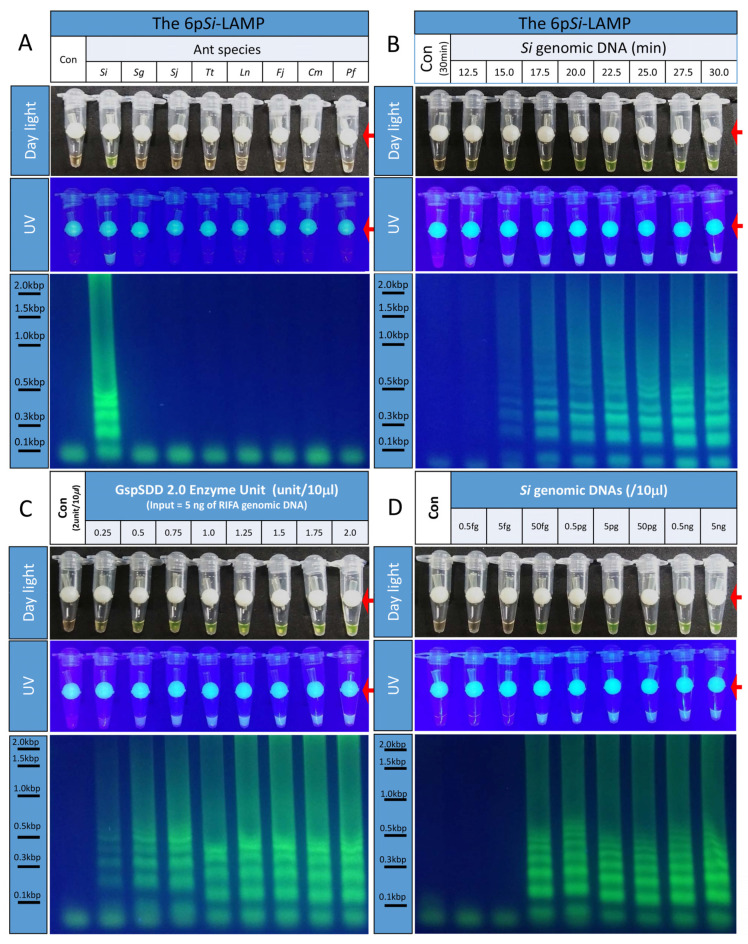
Identification of optimal conditions for 6p*Si*-LAMP assays. (**A**) When 6p*Si*-LAMP assays were performed, strong fluorescence signals under daylight or UV light were detected only from a *Si* genomic DNA-containing tube. In addition, LAMP-specific ladder-shaped DNA bands were only detected from a *Si* genomic DNA-containing tube. (**B**) To determine the optimum incubation period for the 6p*Si*-LAMP assay, LAMP mixtures containing 5 ng of *Si* genomic DNA were incubated at 65 °C for 12.5 to 30 min at 2.5-min intervals. Fluorescence signals under daylight or UV illumination were detected after 12.5 min. When the reactions were separated by a 1.5% agarose gel, ladder-shaped DNA bands were detected from tubes incubated longer than 15 min. (**C**) To determine optimal amounts of GspSDD 2.0 DNA polymerases for the 6p*Si*-LAMP assay, various amounts of polymerases were added. Weak or strong fluorescence and ladder-shaped bands began to be detected at 0.25 unit/10 μL or 1.0 unit/10 μL, respectively. (**D**) To determine the detection limits of the 6p*Si*-LAMP assay, various amounts of *Si* genomic DNA were used for 6p*Si*-LAMP assays. Strong fluorescence signals and ladder-shaped DNA bands were detected at concentrations 50 fg/10 μL and above. Red arrows indicate locations of the fluorescence dye-delivering device inside tubes.

The addition of loop primers into LAMP mixtures has been shown to reduce incubation periods [14]. Thus, we first designed a loop forward (LF) and a loop backward (LB) primer and then tested whether six-primer *Si*-specific LAMP (6p*Si*-LAMP) assays were specific to *Si* (Figure 2A). When 6p*Si*-LAMP assays were performed with the genomic DNA of *Si* and seven other ants, strong fluorescence signals were only detected from *Si* genomic DNA. No signal was observed in the Con. When LAMP reactions were separated by a 1.5% agarose gel, ladder-shaped DNA bands were clearly detected (Figure 2A). This result suggested that the 6p*Si*-LAMP assay was specific to *Si*.

### 3.5. Reduced Incubation Periods of the 6pSi-LAMP Assay

To determine whether the addition of two loop primers to the 4p*Si*-LAMP assay could reduce the incubation period, 6p*Si*-LAMP assays with *Si* genomic DNA with different incubation periods were performed (Figure 2B). Very weak or strong fluorescence signals were detected after 12.5 or 15.0 min of incubation, respectively. However, when the reaction mixture was separated with a 1.5% agarose gel, weak or strong LAMP-specific ladder-shaped DNA bands were observed after 15 or 17.5 min, respectively. This indicates that the inclusion of an LF and an LB primer in the 6p*Si*-LAMP assay can reduce incubation times by 5 min compared with the 4p*Si*-LAMP assay.

### 3.6. Determination of the Optimal Amounts of GspSDD 2.0 DNA Polymerase for the 6pSi-LAMP Assay

The optimal amount of GspSDD 2.0 DNA polymerase was determined to further optimize the efficiency of the 6p*Si*-LAMP assay (Figure 2C). The amount of GspSDD 2.0 DNA polymerase contained in the 6p*Si*-LAMP reaction was increased from 0.25 unit/10 μL to 2.0 unit/10 μL at a rate of 0.25 unit/10 μL. When the 6p*Si*-LAMP assay result was examined, a very weak fluorescence signal was observed even in the reaction including 0.25 unit/10 μL under daylight or UV. At the same time, weak ladder-shaped DNA bands were observed when the 6p*Si*-LAMP reaction was separated by 1.5% agarose gel analysis. Strong fluorescence and bands were observed in the reaction with more than 1.0 unit/10 µL. Therefore, the optimal amount of GspSDD 2.0 DNA polymerase for the 6p*Si*-LAMP assay may be 1.0 unit/10 µL.

### 3.7. Determining the Detection Limits for the 6pSi-LAMP Assay

To determine the detection limit of the 6p*Si*-LAMP assay for *Si* genomic DNA, 6p*Si*-LAMP assays containing a range of *Si* genomic DNA concentrations (from 0.5 fg/10 μL to 5 ng/10 μL with 10-fold differences between each concentration) were run and incubated for 30 min (Figure 2D). The lowest amount of *Si* genomic DNA showing strong fluorescence signals and ladder-shaped DNA bands was 50 fg/10 μL, and the fluorescence signals and patterns of ladder-shaped DNA bands observed from the 6p*Si*-LAMP reactions with more *Si* genomic DNA were not significantly different. These results suggested that the 6p*Si*-LAMP assay has very high sensitivity to *Si* genomic DNA.

### 3.8. The 6pSi-LAMP Assays with Si Genomic DNA Rapidly Extracted with Quick Genomic DNA Extraction Solution

**Figure 3:**
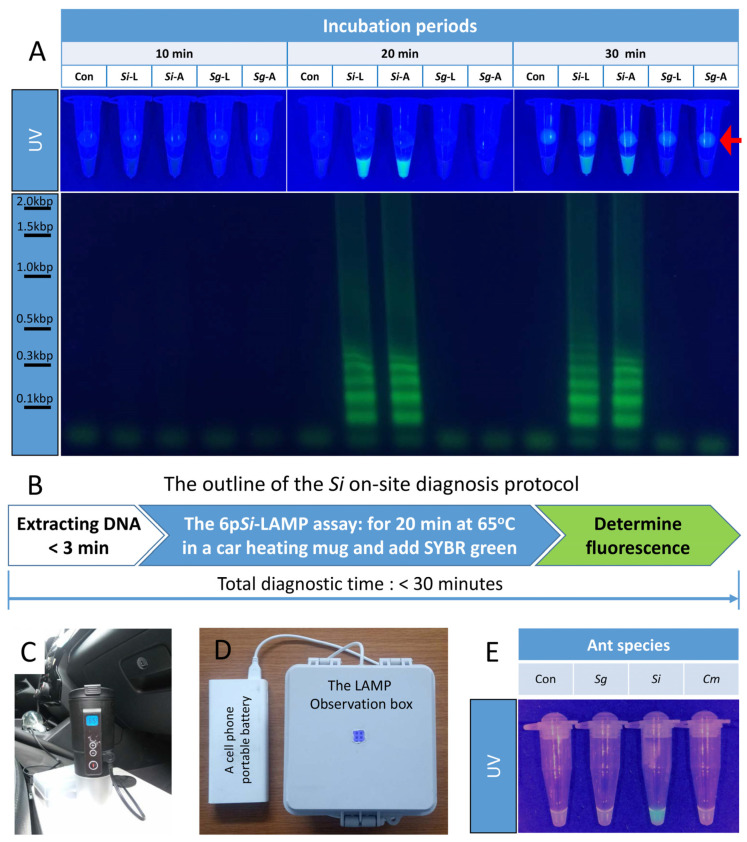
Determination of incubation times for 6p*Si*-LAMP assays and establishment of the *Si* on-site diagnosis protocol. (**A**) When genomic DNA from a leg (L) or an abdomen (A) of *Si* or *Sg* extracted with QGDES, respectively, was used for 6p*Si*-LAMP assays, strong fluorescence signals and ladder-shaped DNA bands were detected only from *Si* genomic DNA after 20 min. (**B**) The outline of the *Si* on-site diagnosis protocol. *Si* can be diagnosed in 30 min on-site. After genomic DNA from an abdomen of *Sg*, *Si*, or *Cm* extracted with QGDES for 3 min was mixed with a 6p*Si*-LAMP assay mixture at a 1:20 ratio, tubes were incubated for 20 min in a car heating mug (**C**). After adding SYBR green to each tube, fluorescence signals were detected in a LAMP observation box powered with a portable cell phone battery (**D**) and photographed with a cell phone (**E**). The red arrow indicated the fluorophore-delivering devices.

The rapid extraction of genomic DNA from ant specimens is essential for 6p*Si*-LAMP assays to be used in the field. When Quick genomic DNA extraction solution (QGDES) was used to extract genomic DNA from a leg or a body segment, 3 min of extraction was sufficient. When 6p*Si*-LAMP assays were performed with genomic DNA extracted from a leg or a body segment of *Si* or *Sg* with QGDES, strong fluorescence signals and ladder-shaped DNA band patterns were observed after 20 min of incubation only from tubes containing *Si* (Figure 3A).

### 3.9. The Si On-Site Diagnosis Protocol

Next, we tested whether genomic DNA extraction with QGDES from ants and 6p*Si*-LAMP assays could be performed in a rest area on the hillside of Mount Suri, Anyang, Gyeonggi-do (Figure 3B–E). The outline of the *Si* on-site diagnosis protocol was depicted in Figure 3B. LAMP reactions were performed with a car heating mug set at 65 °C (C), and a LAMP observation box was used to detect fluorescence signals (D). Strong fluorescence signals were only observed from genomic DNAs extracted with QGDES from *Si*, but not from other ants (Figure 3E). The established *Si* on-site diagnosis protocol can be completed within 30 min from extracting genomic DNA to species identification (Figure 3B).

## 4. Discussion

The major goal of this study was to develop a *Si* diagnostic tool that overcomes the several disadvantages of currently available diagnostics. Our diagnostic tool overcomes several serious shortcomings of current diagnostics. First, molecular diagnostics are needed to minimize the ant specimens required for diagnosis. Among the various molecular diagnostic methods available, the most practical method for on-site diagnostics is a LAMP-based method, which is known to only require an isothermal device and permits the results to be determined by adding ds-DNA intercalating fluorophore to a tube. Thus, the most significant contribution from this study was the identification of a *Si*-specific biomarker suitable for the LAMP assay (Figure 1 and Table 1). Because LAMP assays require at least four to six primer sets, designing specific primer sets for LAMP assays with certain genes or genomic DNA can be difficult. We used *Si*11977, one of two *Si*-specific genes used for the development of the TaqMan RT-PCR methods for the identification of *Si* [4]. Many LAMP assays, including recently published ones for *Si* [12], were developed using COX1 or internal transcribed spacer (ITS) regions that possess certain sequence variants that distinguish *Si* from other related species. In this study, we used *Si*-specific genes to develop the LAMP assay, which was possible because *Si* genome sequences were available [23]. We identified two genes that could be *Si*-specific by performing transcriptome analysis followed by bioinformatics as in a previous study [4]. In addition to *Si*, we have also developed LAMP assays for the detection of *Bursaphelenchus xylophilus* [24], *Drosophila suzukii* [25], and *Spodoptera frugiperda* [26], using species-specific genes or a specific insecticide-resistant allele [27]. Although the development of LAMP assays for species-specific genes requires more research effort than using COX1 or ITS regions, given that the sequences of COX1 or ITS regions are known to often show large differences both within and between species [28], it is possible that invading insects could develop new mutations in COX1 or ITS regions as they adapt and spread. Thus, developing LAMP assays with structural genes such as *Si*11977 in portions of genomic DNA with a lower likelihood of being mutated could produce more reliable and consistent diagnostic results.

The second contribution from this study was the rapidity with which our tool can identify species, in that the 6p*Si*-LAMP assay can diagnose *Si* within 20 min (Figure 2). Previously reported LAMP assays for *Si* require a 90 min incubation period in addition to *Si* genomic DNA extraction and purification [12], which precludes their use in the field.

The third contribution from this study was establishing a rapid *Si* genomic DNA extraction protocol. Currently, various methods for extracting genomic DNA from various tissues, including insects, are available. For example, commercialized genomic DNA extraction kits in this study and from other companies can be used to extract sufficient genomic DNA for various analyses of ants and other insects. However, extracting genomic DNA from insects using these kits requires several steps, such as tissue homogenization, extraction, and final DNA purification, and at least 45 min to one or two days. In this study, we found that *Si* genomic DNA extracted by QGDES (Prime4Dia) for 2 to 3 min from a leg or a body segment was suitable for performing 6p*Si*-LAMP assays without further treatment.

## 5. Conclusions

In summary, we developed a *Si* on-site diagnostic protocol that included the extraction of genomic DNA from captured ant samples in 2 to 3 min and a 6p*Si*-LAMP assay to determine whether a tested ant was *Si* in 20 min. Thus, the total time required for this *Si* on-site diagnostic protocol was less than 30 min from genomic DNA extraction to final diagnosis, which is similar to the time required for currently commercialized RLDAs (Agdia).

## 6. Patents

Patent Applicant—Industry Academic Cooperation Foundation, Hallym University; Inventor: A-Young Kim and Young Ho Koh; Patent Title—Molecular diagnostic method to quickly and accurately diagnose red imported fire ants, *Solenopsis invicta*, in the laboratory and field using Loop-mediated isothermal amplification. Patent Application No. 2021-0116903, Filing date; 2 September 2021, Republic of Korea.

## Figures and Tables

**Table 1:** Oligonucleotide sequences of six primers for the *Si*11977 LAMP assay.

Name	Nucleotide Sequences
*Si*11977-F3	CTAAAACCCCACGCCACTCACC
*Si*11977-B3	TTCGGAGAAGTTGCCGACAATGG
*Si*11977-FIP	CGGGTTTCCTTTGTTTGGGGGACGCAACCACCGTTCATC
*Si*11977-BIP	AAGACACTTTCGTCCCTGCGCTCGCACATACACTTGCATCG
*Si*11977-LF	GATCCCGTCTCAGTCGTCGAT
*Si*11977-LB	ATCCTGACGAGGCCCATTC

## Data Availability

Data available within the article.
